# Technical and dosimetric implications of respiratory induced density variations in a heterogeneous lung phantom

**DOI:** 10.1186/s13014-018-1110-2

**Published:** 2018-09-04

**Authors:** Dennis J. Mohatt, Tianjun Ma, David B. Wiant, Naveed M. Islam, Jorge Gomez, Anurag K. Singh, Harish K. Malhotra

**Affiliations:** 1 Medical Physics Program, Jacobs School of Medicine and Biomedical Sciences, University at Buffalo, Buffalo, NY 14214-3005 USA; 2 Department of Radiation Medicine, Roswell Park Cancer Institute, Buffalo, NY 14293 USA; 3 Radiation Oncology, Cone Health Cancer Center, Greensboro, NC 27403 USA

**Keywords:** Lung SBRT, Acuros XB, Respiratory induced tissue heterogeneity, Phase weighted density

## Abstract

**Background:**

Stereotactic Body Radiotherapy (SBRT) is an ablative dose delivery technique which requires the highest levels of precision and accuracy. Modeling dose to a lung treatment volume has remained a complex and challenging endeavor due to target motion and the low density of the surrounding media. When coupled together, these factors give rise to pulmonary induced tissue heterogeneities which can lead to inaccuracies in dose computation. This investigation aims to determine which combination of imaging techniques and computational algorithms best compensates for time dependent lung target displacements.

**Methods:**

A Quasar phantom was employed to simulate respiratory motion for target ranges up to 3 cm. 4DCT imaging was used to generate Average Intensity Projection (AIP), Free Breathing (FB), and Maximum Intensity Projection (MIP) image sets. In addition, we introduce and compare a fourth dataset for dose computation based on a novel phase weighted density (PWD) technique. All plans were created using Eclipse version 13.6 treatment planning system and calculated using the Analytical Anisotropic Algorithm and Acuros XB. Dose delivery was performed using Truebeam STx linear accelerator where radiochromic film measurements were accessed using gamma analysis to compare planned versus delivered dose.

**Results:**

In the most extreme case scenario, the mean CT difference between FB and MIP datasets was found to be greater than 200 HU. The near maximum dose discrepancies between AAA and AXB algorithms were determined to be marginal (< 2.2%), with a greater variability occurring within the near minimum dose regime (< 7%). Radiochromatic film verification demonstrated all AIP and FB based computations exceeded 98% passing rates under conventional radiotherapy tolerances (gamma 3%, 3 mm). Under more stringent SBRT tolerances (gamma 3%, 1 mm), the AIP and FB based treatment plans exhibited higher pass rates (> 85%) when compared to MIP and PWD (< 85%) for AAA computations. For AXB, however, the delivery accuracy for all datasets were greater than 85% (gamma 3%,1 mm), with a corresponding reduction in overall lung irradiation.

**Conclusions:**

Despite the substantial density variations between computational datasets over an extensive range of target movement, the dose difference between CT datasets is small and could not be quantified with ion chamber. Radiochromatic film analysis suggests the optimal CT dataset is dependent on the dose algorithm used for evaluation. With AAA, AIP and FB resulted in the best conformance between measured versus calculated dose for target motion ranging up to 3 cm under both conventional and SBRT tolerance criteria. With AXB, pass rates improved for all datasets with the PWD technique demonstrating slightly better conformity over AIP and FB based computations (gamma 3%, 1 mm). As verified in previous studies, our results confirm a clear advantage in delivery accuracy along with a relative decrease in calculated dose to the lung when using Acuros XB over AAA.

## Background

Stereotactic Body Radiotherapy (SBRT) is an ablative dose delivery technique which requires the highest levels of precision and accuracy [1, 2]. Modeling dose to a lung treatment volume has remained a complex and challenging endeavor for two major reasons. First, the gross tumor volume (GTV) is typically surrounded by lung tissue approximately 75% less dense than the tumor itself [3]. Second, the actual density of the treatment volume is further complicated by the movement of GTV due to patient respiratory motion. Even though certain motion management techniques [4] such as deep inspirational breath hold [5], abdominal compression [6], tumor tracking [7] and respiratory gating [8] have been incorporated with lung SBRT to restrict the size of the irradiated target volume, an additional margin to account for set-up errors is still required which encompasses a substantial portion of low density lung tissue [9]. When coupled together these factors give rise to pulmonary induced tissue heterogeneities which could possibly lead to inaccuracies in dose computation [10].

The accuracy of the dose distributions predicted by the treatment planning system is of critical importance in maximizing the tumor control probability in lung SBRT [11, 12]. Tissue heterogeneity is of particular interest for dose computation in the lung due to the relative low electron density which requires greater photon fluence to achieve a similar build-up equilibrium equivalent to that of soft tissue. Compared to homogeneous media, where modeling of high energy photon beams is a relatively straight forward process, energy transport in heterogeneous media involves an intricate extrapolation of various density-dependent correction factors. To date, the most accurate dose computational algorithm for handling highly heterogeneous media is Monte Carlo (MC) simulation [13] –unfortunately it requires the greatest processing time [14]. For enhanced computational performance, alternative algorithms [15] such as Analytical Anisotropic Algorithm (AAA) [16] and Acuros External Beam (AXB) [17] have been commercially developed which implement various levels of simplifications and assumptions to allow calculations to be completed within clinically acceptable time frames. Fundamentally, material density with AAA is accounted for anisotropically by the implementation of Gaussian weighted photon scattering kernels [18]. In contrast, AXB seeks a more direct approach in solving the linear Boltzmann transfer equation by taking into consideration the specific chemical composition of the surrounding media [19]. Still, dose computational accuracy in all respects is ultimately governed by the actual target density as defined by the CT dataset.

Model based algorithms provide a realistic generation of absorbed dose in heterogeneous media by sampling CT values (Hounsfield Units –HU). This information is then used to scale high energy particle interactions with respect to actual physical density. In modern radiology, 4D computed tomography (4DCT) has become the standard to account for changes in patient anatomy during respiration [20, 21]. Generally, the most common CT datasets used for dose computation for lung SBRT are average intensity projection (AIP) and free breathing (FB). However, FB is essentially a single phase snapshot of the GTV at a given location in time which does not capture the effects of target movement, whereas AIP will compress all temporal motion information into a single 3DCT image. While AIP imaging assigns the average pixel value to a specific location, we also considered the maximum intensity projection (MIP) in this study which assigns only the greatest point pixel value to represent our highest target density scenario. In addition, we introduced a fourth dataset based on a novel phase weighted density (PWD) technique. With the PWD approach, sub-regions generated by 10 individual phased GTV structures were overridden with specific CT values based on the temporal dependence of the GTV overlap.

Despite all state-of-the-art advances in radiological imaging, the medical physics community has yet to reach a consensus to best account for dynamic target motion in heterogeneous media. To date, a limited number of studies have been conducted to compare different image generated datasets [22–27]. However, no systematic study exists to determine which is better for dose computation. Although Monte Carlo simulation is recognized as the gold standard for handling tissue heterogeneity, its use in the clinical environment has been very limited. This investigation aims to determine which combination of imaging techniques and algorithms yields the most accurate dose distributions, under extreme lung density variations, that are experimentally achievable within a clinical environment.

In this study, we evaluate the physical properties associated with motion-induced lung target densities by comparing representative image sets. In addition, we introduce an innovative PWD dataset based on the time dependent location of the GTV structure over the course of one respiratory cycle. We compared each plan calculated for AAA and AXB, and evaluated these dose distributions with respect to the actual dose delivered using radiochromatic film. To ensure our assessment certainty, end-to-end testing of all plans was generated using one treatment planning system, and then delivered using a single Truebeam STx linear accelerator.

## Methods

**Fig. 1:**
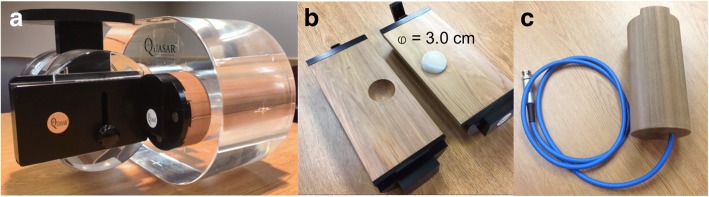
**a** Quasar phantom with (**b**) cedar lung insert encapsulating an offset polystyrene target 3.0 cm diameter. **c** Shows an identical cedar insert with imbedded 3.0 cm target bored out to fit a 0.125 cc ion chamber at isocenter

Lung tumor motion was simulated using a Quasar Respiratory Motion Phantom (Modus, London, Ontario, CA) as shown in Fig. 1. To replicate clinically relevant tissue properties, the phantom contains a low density cedar wood insert that mimics lung tissue (HU = − 750 – -600). This encapsulates an offset spherical polystyrene target (HU = − 100 – 0) 3.0 cm in diameter. The Quasar apparatus provided simple harmonic motion along the superior-inferior direction for which the target range was adjusted to ±0.5, ±1.0, and ± 1.5 cm translational increments at 15 cycles per minute.

### Calculations in anthropomorphic phantom

**Fig. 2:**
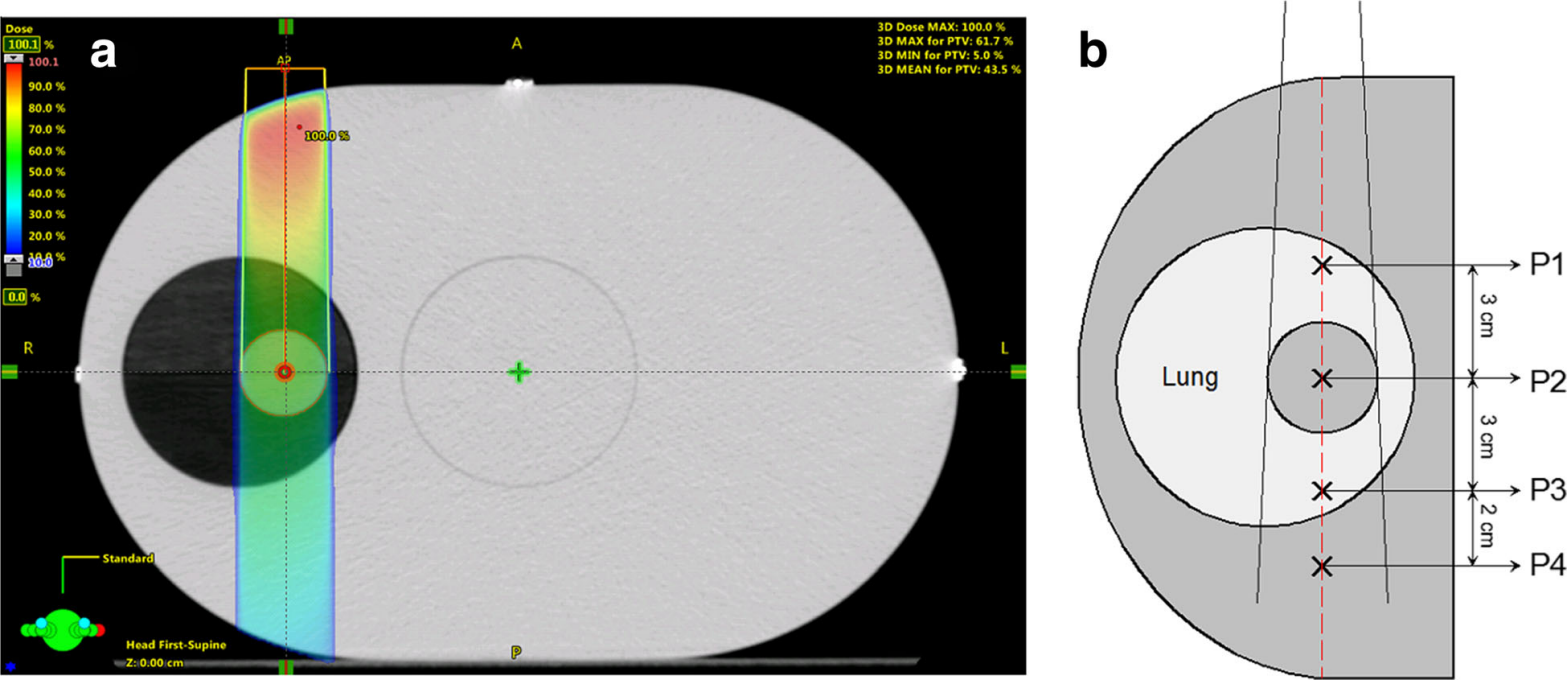
Axial view of (**a**) CT of Quasar phantom, and (**b**) schematic diagram of phantom illustrating dose sampling points of interest for AAA and AXB computations

Heterogeneous density calculations were performed using the Quasar Phantom in a static configuration. A single anterior field (3.0 × 3.0 cm^2^) was aligned perpendicular to the phantom and prescribed with fixed monitor units of 500 MU for photon energy of 6 MV flattening filter free as shown in Fig. 2a. Dose distributions along the beam central axis, as function of depth, were then calculated using AAA and AXB algorithms with 0.2 cm^3^ grid size. As illustrated in Fig. 2b, points of interest were selected along the central axis where P1 is located 3 cm anterior to the target, P2 at the target isocenter, P3 is 3 cm posterior to the target center, and P4 beyond the lung cavity located 2 cm below P3. To mimic density variation effects caused by tumor movement, the CT values of the target structure were systematically overridden from − 200 to + 200 in increments of 25 HU. Due to the density variation of the target structure, a relationship between the dose errors was represented as percentage dose change relative to nominal HU value of the target for each point of interest.

### 4DCT imaging and target delineations

4DCT image sets were acquired using a GE Lightspeed Pro 16 slice scanner (General Electric, Milwaukee, WI), with a slice width setting of 1.25 mm. Respiratory cycle information was subsequently recorded using a Real-time Position Management (RPM) system (Varian, Palo Alto, CA). In general, the external movement of the phantom was synchronized to the internal movement of the target via an illuminated infrared signal reflected from a marker block and directed to a CCD camera. The recorded respiratory waveform was then referenced in the binning process to coordinated projected image sets, with respect to their appropriate anatomical locations, over the course of one respiratory period. Reconstructed images and respiratory data were then transferred to Advantage 4D workstation (General Electric, Milwaukee, WI). These images were sorted and binned with respect to couch position and corresponding respiratory phase at ten uniformly spaced intervals within the respiratory cycle, with CT_0%_ indicating the max-inhalation phase and CT_50%_ the max exhalation phase. From these bins, AIP and MIP image sets were automatically generated by selecting the average and the maximum pixel densities across all respiratory phases of the 4DCT dataset, respectively. A subsequent helical image was also taken immediately after the 4DCT acquisitions and was designated as the corresponding free-breathing (FB) scan. This procedure was repeated for target amplitudes ranging from ±0.5 to ±1.5 cm at ±0.5 cm increments, for which the processed images were imported into Eclipse (version 13.6) treatment planning system (TPS). Furthermore, an additional FB scan was taken with the no phantom motion and planned accordingly for verification of image registration and dose calibration process.

In order to infer the motion information using Eclipse, manual contouring of individual GTVs was performed on individual CT image sets within multiple respiratory phases (CT_0%_ - CT_90%_). To avoid any interplanner differences, all contours were segmented by one individual using the same lung window setting in all image sets. The internal target volume (ITV) structure was then generated using Boolean “OR” operation to union all 10 GTV phased structures from individual CT image sets corresponding to set motion amplitude. In accordance with RTOG 0915 protocol, the planned treatment volume (PTV) was then created by expanding a uniformly isotropic 0.5 cm margin from the ITV [9]. All ITV and PTV structures were initially created in the FB image set and subsequently copied to the AIP, MIP and PWD datasets.

It is noted in a similar study, the Mid-Ventilation (CT_50%_) image set was included for comparative analysis [25]. With the exception of target location, the target density associated with CT_50%_ is very similar to that represented by the FB image set. Therefore, since the central focus of this current study is to evaluate the extreme density variations between datasets, examination of CT_50%_ was not taken into consideration in order to circumvent redundancy.

### Defining the phased weighted density structure

**Fig. 3:**
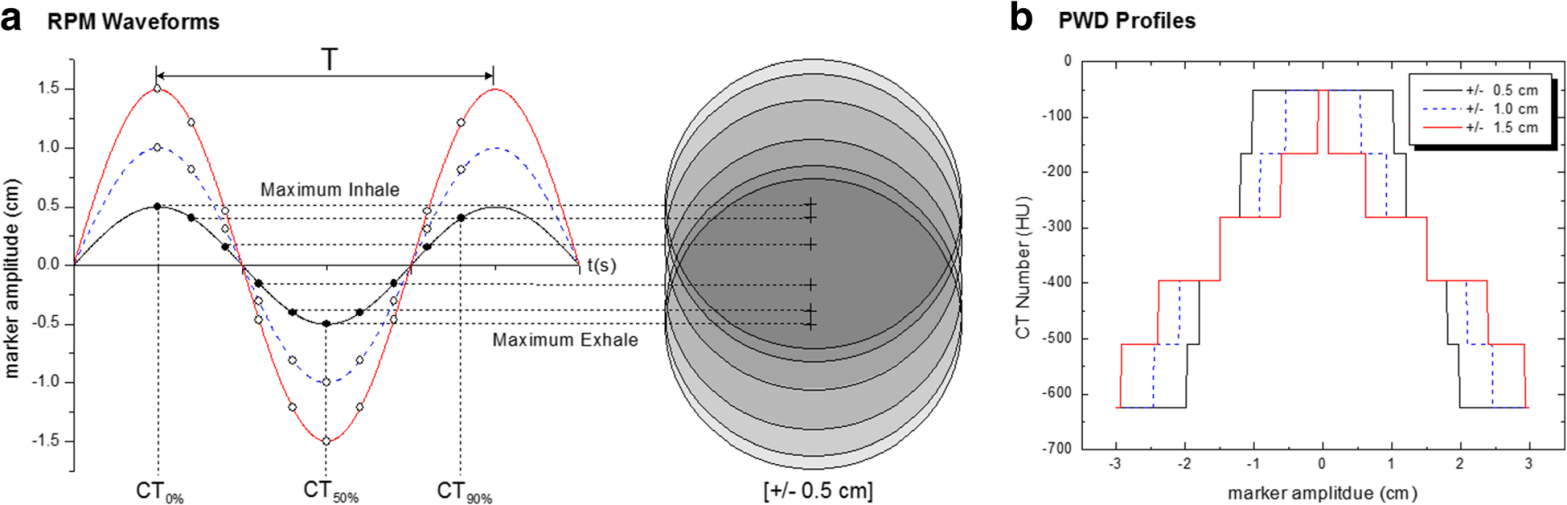
**a** Schematic of respiratory waveform showing the correlation of individual GTV locations. The overlap of the 10 phase GTV contours gives rise to the sub-regions within the phase weighted density (PWD) structure. **b** PWD profiles along the superior/inferior direction showing the HUs as function as marker amplitude

In addition to the FB, AIP and MIP CT datasets, we created a hybrid phase weighted density (PWD) structure for comparison. In principle, the changing density of the target region over time can be generalized by the following relation:

1 $$ d\boldsymbol{\rho} =\left({\boldsymbol{\rho}}_{\boldsymbol{GTV}}+{\boldsymbol{\rho}}_{\boldsymbol{lung}}\right)\boldsymbol{dt} $$

where 흆_GTV_ is the target density, and 흆_lung_ represents the density of the lung. It follows that a solution for the density for a PWD structure (흆_PWD_) yields the following expression

2 $$ {\boldsymbol{\rho}}_{\boldsymbol{PWD}}=\left(\frac{\boldsymbol{t}}{\boldsymbol{T}}\right){\boldsymbol{\rho}}_{\boldsymbol{GTV}}+\left(\frac{\left(\boldsymbol{T}-\boldsymbol{t}\right)}{\boldsymbol{T}}\right){\boldsymbol{\rho}}_{\boldsymbol{lung}} $$

where t is the occupational time of the GTV at one location, and T is the period of the respiratory cycle. In practice, the duration of t occurs at discrete increments of 1/10th of the respiratory period in association with the phase binning process. Thus, as illustrated in Fig. 3, the final t value reflects how many times the GTV has overlapped with itself during the period of oscillation, giving rise to higher density within a particular sub-region.

Discrete sub-regions within the ITV were created by the addition and/or subtraction of the 10 individually defined GTV phase binned structures, using Boolean operations within the TPS. CT values for each sub-region were then overridden and weighted accordingly to the mean GTV (− 50 HU) and lung (− 625 HU) density value as defined by the FB image set.

### HU density extraction

**Fig. 4:**
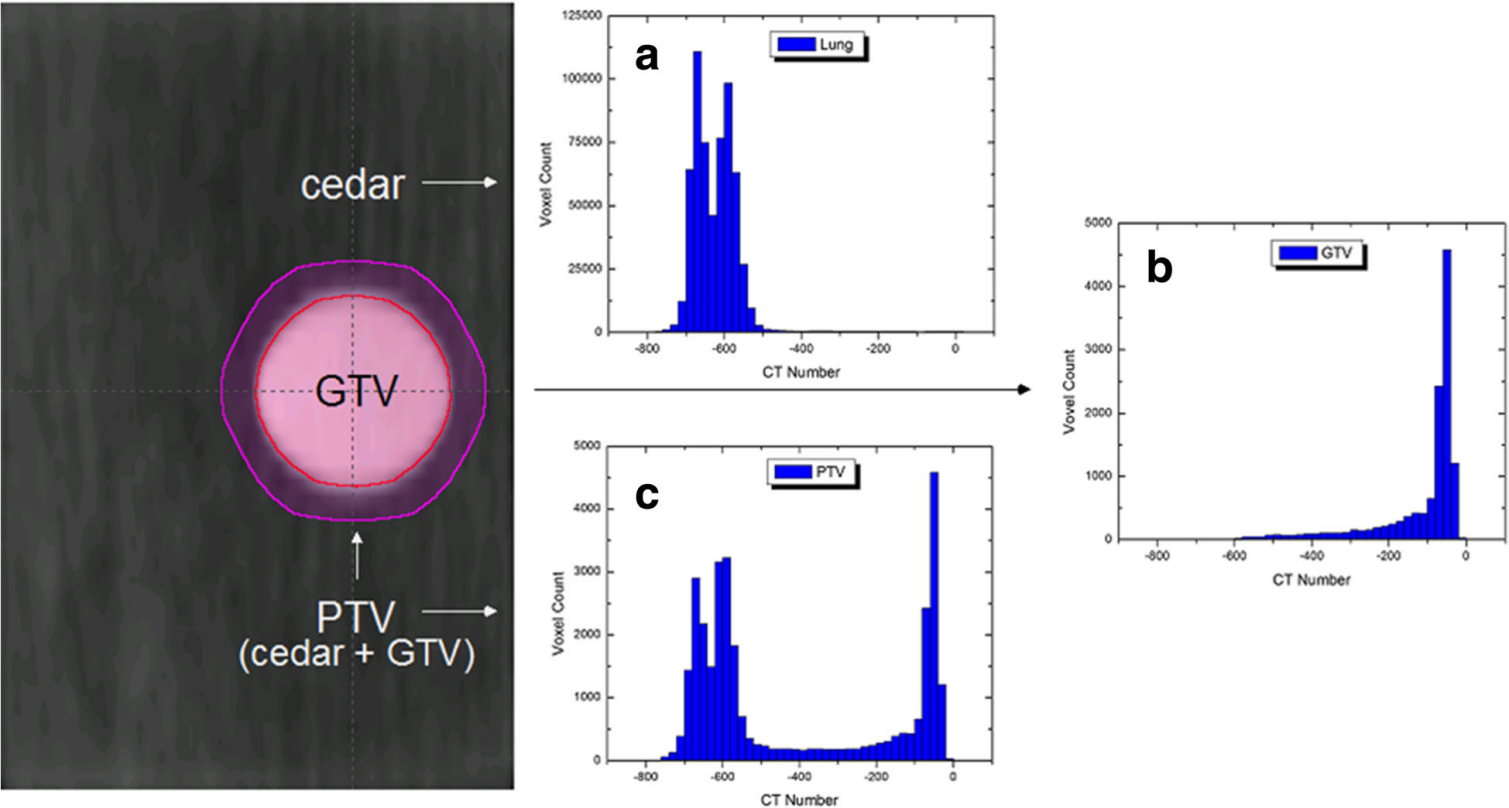
Frontal view of target structure and extracted HU information. Histograms represent the relative density of the **a** cedar insert, **b** polystyrene target (GTV), and **c** the planned treatment volume (PTV) which includes a 5 mm isotropic margin around the GTV

HU voxel values for each PTV structure were extracted using an in-house program written in Matlab (MATLAB, The MathWorks Inc., Natick, MA, 2017). For FB, AIP and MIP data set, original DICOM CT images and RT structures were directly imported into Matlab software. Based on the corresponding contoured structure set, a binary mask was created to segment out the region of interest (ROI) for HU data export. The output of all HU values inside the ROI for each image set were then analyzed as a histogram format ranging from CT numbers − 850 to 50 HU with bin size of 20. Plotting and statistical analysis was performed using Origin (version 6.0) software. Using the HU density extraction method, Fig. 4 shows the histogram representations for the (a) cedar insert, (b) polystyrene target, and (c) PTV structure.

### Treatment planning

Individual treatment plans were created from FB, AIP, MIP and PWD datasets in Eclipse. Irradiation of the target structure was planned for a photon 6 MV flattening filter free beam using simple Anterior-Posterior (AP) and Posterior-Anterior field configurations. The MLC was fitted to the shape of the PTV and collimator jaws were set to the recommended position determined by the TPS, with width spacing no less than 3.5 cm as dictated by RTOG protocol [9]. Dose calculations for each plan were executed using AAA and AXB algorithms at a predetermined value of 500 MU per field for 0.2 cm grid size. The initial parameters for segmented ITV and PTV structures for each range of motion are summarized in Table 1.

**Table 1:** A summary of the initial parameters for all ranges of target motion

Range of motion (cm)	ITV (cc)	PTV (cc)	Rx Dose (MU)
1 [± 0.5]	21.87	48.41	500
2 [± 1.0]	27.36	58.33	500
3 [± 1.5]	34.64	71.13	500

### Radiochromatic film measurements

Phantom set up was done using the same simulation positions for dose delivery by means of a TrueBeam STx. The dose from the treatment planning system was verified using an identical cedar insert phantom (Modus, Model No. 500–3332) specifically designed to house a PTW 0.125 cc ion chamber (Fig. 1c). Gafchromic EBT3 film (International Specialty Products, Wayne, NJ, Lot #: 03311401) was used for both film calibration and phantom measurements. The film was positioned inside the cedar insert, central to the target location and marked at the time of irradiation. Each phantom irradiation measurement was repeated 3 times for which subsequent measurements were taken to reference machine output and scaled to film response. The film was then stored in a dry, dark environment for 24 h and later scanned using Epson Perfection V700 flatbed scanner (Epson America, Inc. Long Beach, Ca) with 48 bit color and 150 dpi resolution. RIT113 (Radiological Imaging Technology, Inc., Colorado Springs, CO) version 5.1 software was used to analyze the film using the red channel. A dose calibration curve for the red channel was generated by irradiating individual films for known doses from 0 to 10 Gy.

Due to phantom motion, the center of film does not remain in the center of dose distribution over the time of delivery. Hence, dose generated from a static CT by the TPS cannot be directly compared to the dose measured on moving film (consult Ref [26] for an excellent review of the dose smearing effect and compensation). Film motion was accounted for by convolving the TPS dose using a custom script written in MATLAB developed by Wiant et al. [26]. Convolved dose plane distributions were then imported into RIT113 software for gamma analysis. As formulated by Low, the standard criterion for “measured” versus “calculated” dose was evaluated for a particular dose threshold within an acceptance radius (i.e. 3%, 3 mm) [28].

## Results

### Anthropomorphic lung phantom calculations

**Fig. 5:**
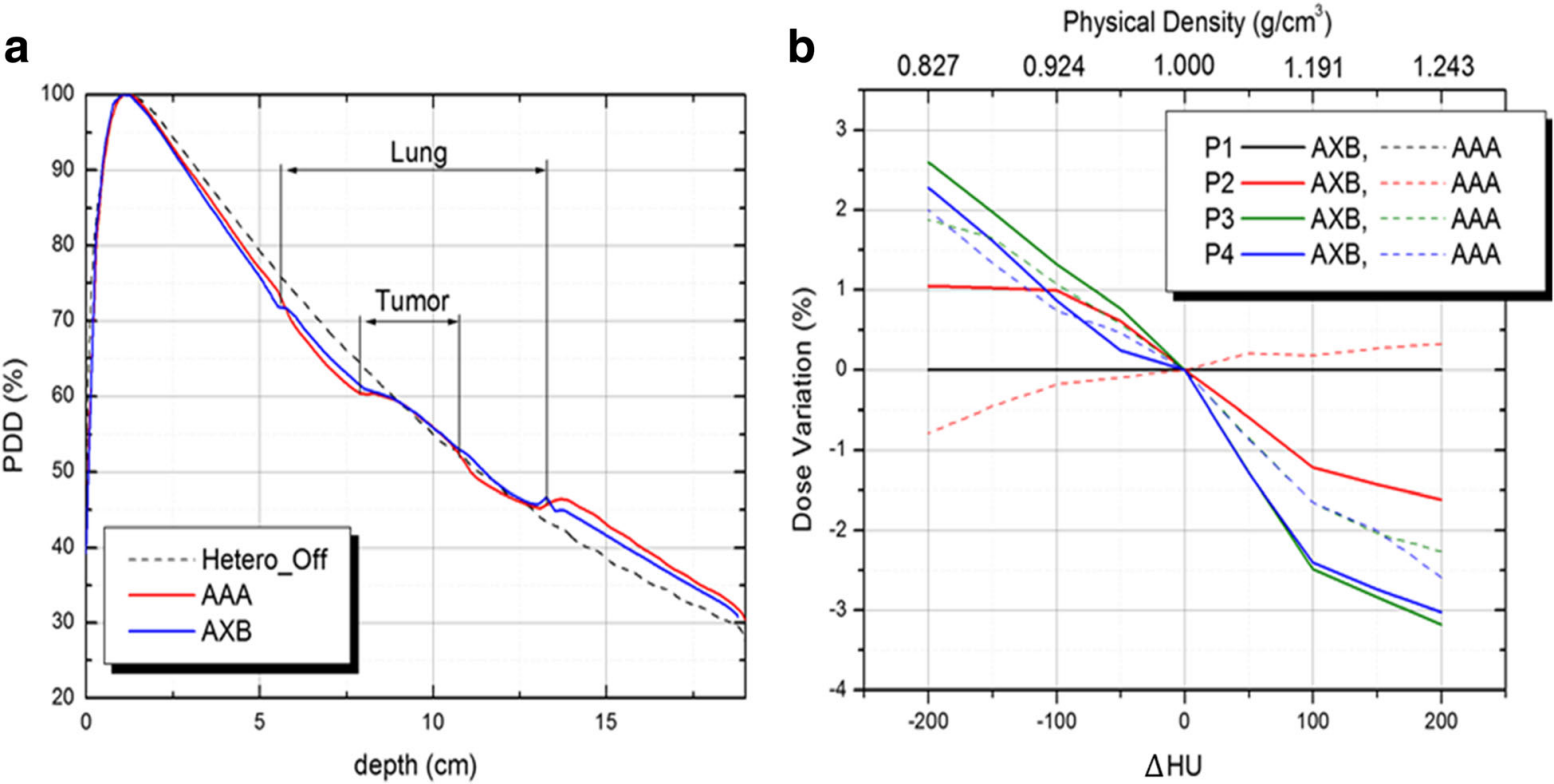
Results of phantom calculations for profiles and point taken along the central beam axis. **a** Percent depth dose curves for AAA and AXB as compared to a calculation with no heterogeneity corrections. **b** Dose errors at particular points of interest relative to the nominal CT value of the GTV structure. Different colors are used to represent P1, P2, P3 and P4 points of interests as defined in Fig. 2b. Solid versus dashed traces are used to distinguish Acuros XB versus AAA calculations, respectively

The results of our phantom calculations in a static configuration are shown in Fig. 5a, b. For a single AP field as shown in Fig. 2a, the percent depth dose (PDD) profiles for AAA and AXB are plotted in Fig. 5a. When compared to calculations with no heterogeneity, both the AAA and AXB profiles are virtually identical as indicated by the dashed line. The algorithms reveal a subtle distinction between lung versus tumor media, which is unnoticeable when heterogeneity is turned off. Interestingly, the first time these curves intersect after the build-up region, is near isocenter of the target. This is where the target density is roughly equivalent to that of water.

Figure 5b shows the trend in dose errors at particular points of interest in reference to Fig. 2b. For each point, the percentage dose error is defined relative to the nominal CT value of the GTV structure, which was overridden spanning from − 200 to 200 HU. For the fixed point located just above the target (P1), both AAA and AXB traces are flat lined and unaffected by the downstream change of target density. At isocenter (P2), the negative slope associated with AXB calculation indicates the relative increase in target density, or photon attenuation. This attenuation is compensated for by the decrease of photon fluence. On the other hand, the slightly positive slope associated with the AAA algorithm indicates an opposing effect caused by secondary electron transport. As interpreted in a related study by Liu, the density variation of the target when using AAA makes a larger impact on electron fluence over photon attenuation, where electron fluence becomes the dominating factor for compensation [29]. For points beyond the target structure, both algorithms show a similar attenuation response to the target density variation within the lung (P3), and beyond (P4) where electron equilibrium is re-established.

### Extracted PTV density formations

**Fig. 6:**
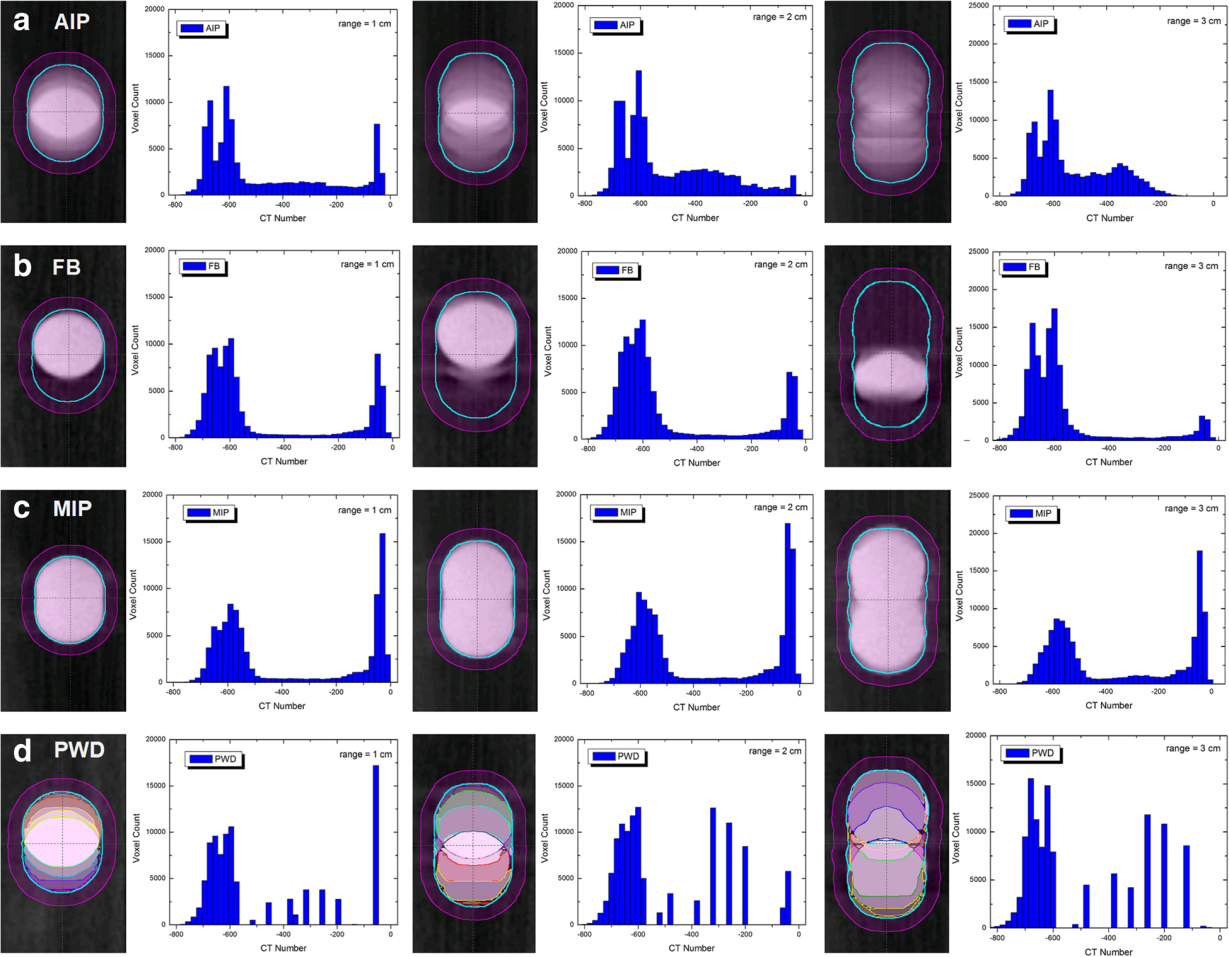
Histogram density representations for **a** AIP, **b** FB, **c** MIP and **d** PWD target structures ranging from 1 to 3 cm of motion. In general, as range of motion increases the average CT value within the PTV decreases for all datasets

The wide range of density variations for each PTV structure is illustrated in Fig. 6. As shown from left to right, each column represents an additional 1 cm increase in target movement. Each row displays voxel count versus CT number as defined by the AIP, FB, MIP and PWD data sets. Due to the 5 mm isotropic margin expansion from the ITV, the PTV will include a significant portion of low density media concentrated at approximately − 625 HU. In general, as the range of target movement increases, the GTV peak (center around − 50 HU) will essentially become absorbed into the lower density media. This effect is most pronounced for FB data sets, where the average CT number decreases from − 479 to − 569 HU, versus AIP − 473 to − 524 HU, and PWD − 472 to − 507. For MIP data sets, the average CT number remains fairly consistent ranging from − 352 to − 370 HU. Thus, in our most extreme case scenario, the difference between low density FB and high density MIP data sets is greater than 200 HU. The PTV average CT values extracted from each dataset are listed in Table 2.

**Table 2:** The mean HU values per PTV and percentage difference for dosimetric parameters between AXB vs AAA calculations. For 1–3 cm superior-inferior target motion, the dose difference for FB, AIP, MIP and PWD datasets is given via $\Delta  D=\left(\frac{D_{AXB}-{D}_{AAA}}{D_{AXB}}\right)x100\%$

Target range	CT dataset	<HU > ±sd	∆D_max_	∆D_2%_	∆D_mean_	∆D_95%_	∆D_98%_
1 cm	AIP	-473 ± 223	−1.40	−1.40	0.06	−4.63	−4.80
	FB	−479 ± 246	−1.40	− 1.34	0.03	−4.39	− 5.22
	MIP	−370 ± 266	− 1.82	− 2.17	− 0.09	− 4.49	−4.79
	PWD	−472 ± 239	− 2.12	−2.52	− 0.33	−2.37	− 3.34
2 cm	AIP	− 503 ± 178	− 1.75	− 1.31	− 0.08	−5.07	− 5.43
	FB	−507 ± 227	− 0.91	− 0.50	0.23	− 4.92	− 6.73
	MIP	− 356 ± 259	− 1.91	− 2.15	−0.36	− 4.52	− 4.32
	PWD	− 490 ± 207	−1.40	− 1.37	−0.06	− 4.26	− 5.43
3 cm	AIP	−524 ± 143	−1.54	− 1.16	−0.23	−5.64	− 6.68
	FB	−569 ± 178	− 0.72	− 0.80	0.20	−3.26	− 3.71
	MIP	− 352 ± 249	− 2.14	−2.10	−0.62	−5.42	− 6.91
	PWD	−507 ± 208	− 1.98	− 1.87	− 0.37	−4.68	−5.28

### AAA vs. AXB Dosimetric impact

Results for mean PTV HU values and dosimetric differences between AXB versus AAA are listed in Table 2. In this analysis we considered the dosimetric parameters (D_max_, D_2%_,, D_mean_, D_95%_, D_98%_) as evaluated from dose volume histograms (DVH). When compared with AXB, AAA will consistently overestimate the dose to the treatment volume. This is indicated by a negative sign for a predominant portion of the analysis with exceptions occurring at D_mean_ (D_50%_) –the approximate location of where the two curves may intersect. In general, the near maximum dose (D_2%_) discrepancies are marginal (< 2.2%), with a greater variability occurring within the near minimum (D_98%_) dose regime (< 7.0%). Considering that the size of the PTV structure increases as range of motion increases (see Table 1), the resulting widening of collimator jaws will yield a slight increase in dose at target isocenter. This can be depicted by the standard deviation. Thus, even for extended range of target motion, the dose discrepancies between the two algorithms near target isocenter are still small, with the greatest differences observed for MIP (2.1 ± 0.1%), followed by PWD (1.9 ± 0.6%), AIP (1.3 ± 0.1%), and least with FB (0.9 ± 0.4%).

**Fig. 7:**
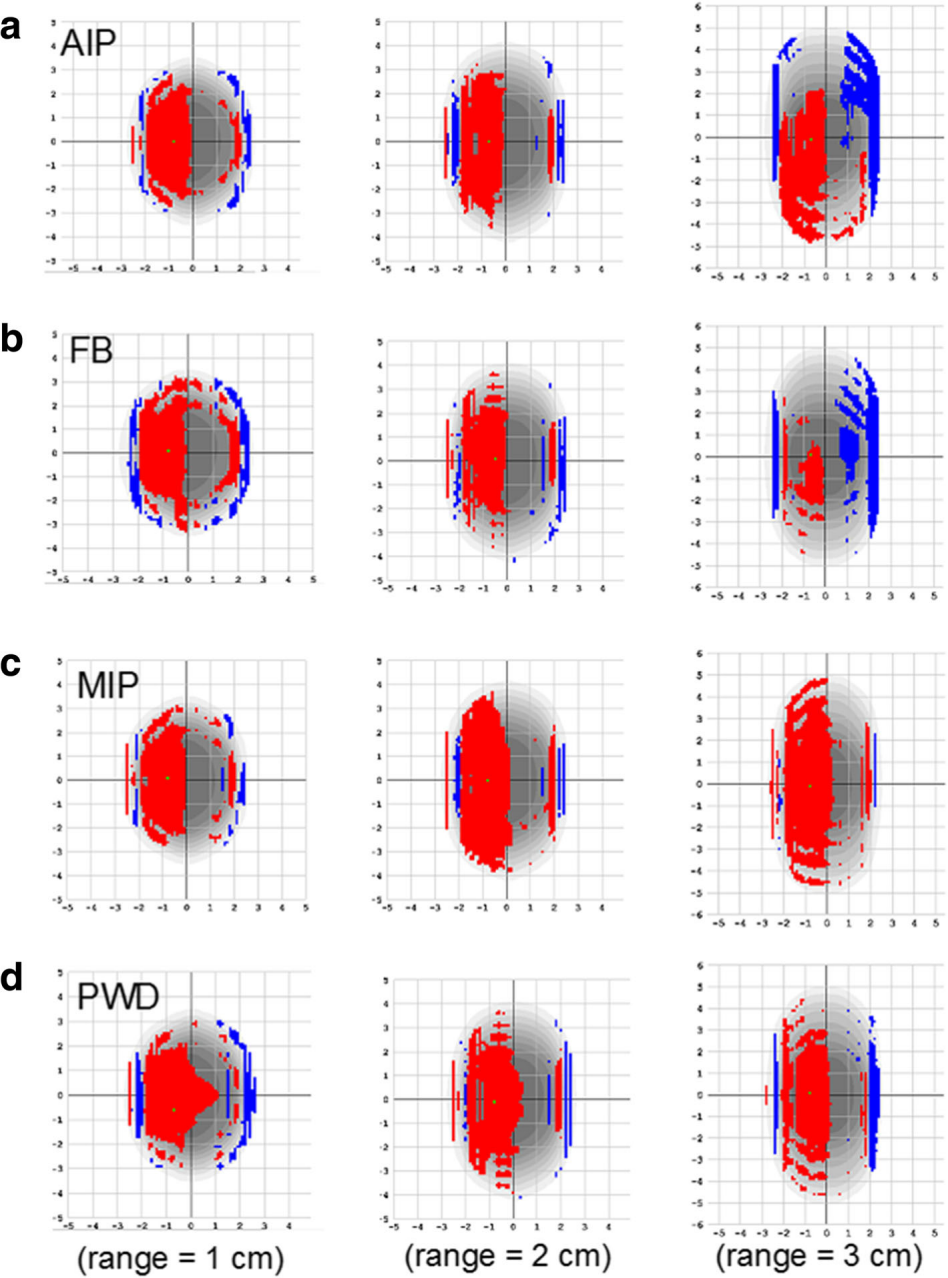
A planner view of the dose distribution mismatch between AAA versus AXB computations. Plans were calculated for **a** AIP, **b** FB, **c** MIP, and **d** PWD data sets for target range of motion spanning from 1 to 3 cm (left to right). The analysis shown here is based on stringent gamma criteria (1%, 0 mm), where regions in red are indicating higher AAA dose calculation, versus blue for lower dose as with respect to AXB

A direct comparison of AAA versus AXB calculated planner dose distributions are illustrated in Fig. 7. Plans generated using the AIP, FB, MIP and PWD image sets for all ranges of motion are benchmarked with respect to AXB-based computations. Using nominal gamma criteria of 3%, 3 mm, all plans are virtually identical with pass rates of 100%. However, when switching to a most stringent criteria of 1%, 0 mm, the gamma index is anywhere from 73.2–54.0%. This predominantly high region of failure is occurring on the left side of the dose distribution in each case, and is caused by a relatively larger cedar gap in the phantom geometry (see Fig. 2b). Hence, in the lower density region the AAA calculation is overestimating the dose to the target, while the AXB algorithm compensates for photon attenuation of the target itself. Likewise, the greater overall discrepancies are generally occurring with the higher density MIP datasets.

### Radiochromic film verification

**Fig. 8:**
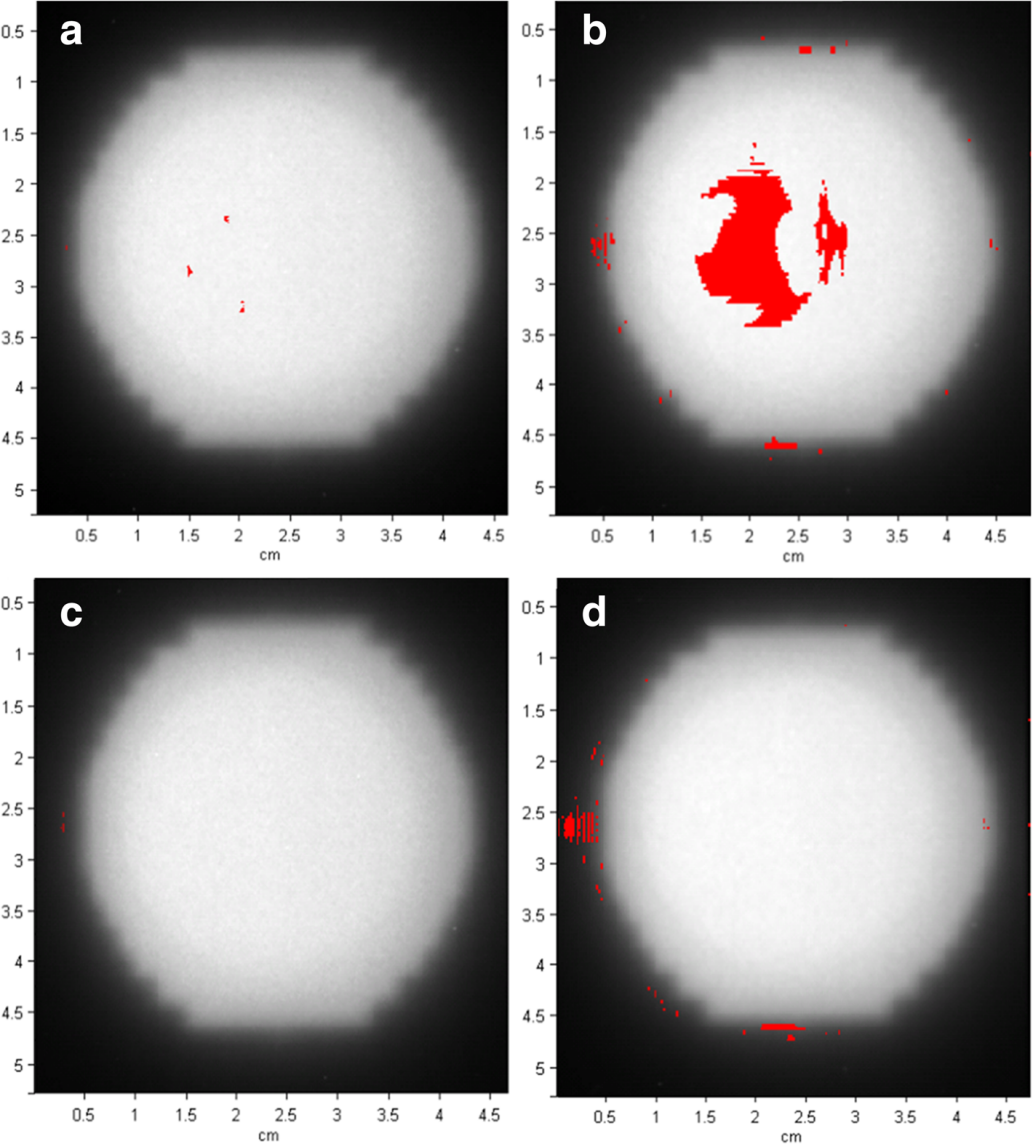
Gafchromic film verification of image registration and dose calibration. Gamma pass rates for: **a** AAA (3%,3 mm) 99.0 ± 1.5%, **b** AAA (3%,1 mm) 91.1 ± 3.8%, **c** AXB (3%,3 mm) 99.7 ± 0.2%, and **d** AXB (3%,1 mm) 95.7 ± 3.6%. The red indicate failed pixels for which the gamma index is greater than 1 [28]

All plans were measured using an identical lung insert phantom modified for PTW 0.125 cc ion chamber and were determined to be within < 2% agreement with the treatment planning system. However, these measurements yield only one data point at isocenter. Therefore, gafchromic film analysis was used for complete distribution comparisons for which the verification of the image registration and dose calibration process is shown in Fig. 8. Each static measurement was repeated three times in conjunction with our motion runs, for which the film was analyzed for the same plans calculated for AAA and AXB. These results demonstrate the accuracy and reproducibility for the systematic method of measurement used in this study when no motion was employed. Furthermore, they tend to rule out the inherent uncertainty that may be associated with the differences between both algorithms in question, and suggest the subsequent discrepancies have to do with the density distributions that are represented by the CT datasets once the phantom was sent into motion.

Table 3 summarizes the film results for target motion amplitudes up to ±1.5 cm. Under conventional tolerances (gamma 3%, 3 mm), AIP and FB image sets generated plans with pass rates greater than 98% for both AAA and AXB based computations. When switching to SBRT tolerances (3%, 1 mm), MIP and PWD based AAA computations generally fell below 85% pass rates, and gradually degraded as range of motion increased. However, AXB pass rates for all image sets showed a substantial improvement in delivery accuracy when compared to AAA. Interestingly, the pass rates for the higher density MIP image set yielded comparable results (> 85%) to those of AIP and FB when calculated for AXB algorithm. Although PWD dataset did not perform well for large ranges of motion when evaluated with AAA, PWD demonstrated a slightly better conformance over AIP and FB when using Acuros XB.

**Table 3:** Summary of gamma pass rates (%) for gafchromic film measurements. All plans were measured on 3 separate instances where the error depicts the standard deviation

Range of motion (cm)	CT dataset	AAA		AXB	
		(3%, 3 mm)	(3%, 1 mm)	(3%, 3 mm)	(3%, 1 mm)
1 cm	AIP	99.4 ± 0.4	86.4 ± 5.2	99.8 ± 0.1	88.0 ± 4.1
	FB	98.7 ± 0.7	86.9 ± 5.0	99.8 ± 0.1	88.2 ± 3.6
	MIP	95.9 ± 3.6	80.5 ± 5.7	99.7 ± 0.2	89.8 ± 3.6
	PWD	97.1 ± 3.2	85.7 ± 3.5	99.7 ± 0.2	89.4 ± 5.7
2 cm	AIP	98.6 ± 0.7	85.0 ± 2.3	99.0 ± 0.4	86.8 ± 3.9
	FB	99.3 ± 1.2	87.1 ± 5.6	99.6 ± 0.8	86.8 ± 4.7
	MIP	98.2 ± 0.2	77.8 ± 4.5	99.5 ± 0.8	85.8 ± 4.0
	PWD	98.4 ± 2.5	83.2 ± 4.5	99.6 ± 0.3	89.1 ± 3.6
3 cm	AIP	98.1 ± 1.6	85.4 ± 4.5	99.1 ± 0.8	91.2 ± 1.7
	FB	98.4 ± 1.3	88.8 ± 3.5	99.1 ± 0.8	91.7 ± 4.6
	MIP	94.2 ± 3.8	69.4 ± 5.9	98.9 ± 0.8	88.1 ± 1.5
	PWD	94.1 ± 0.4	76.0 ± 5.2	99.3 ± 0.6	94.2 ± 4.2

**Fig. 9:**
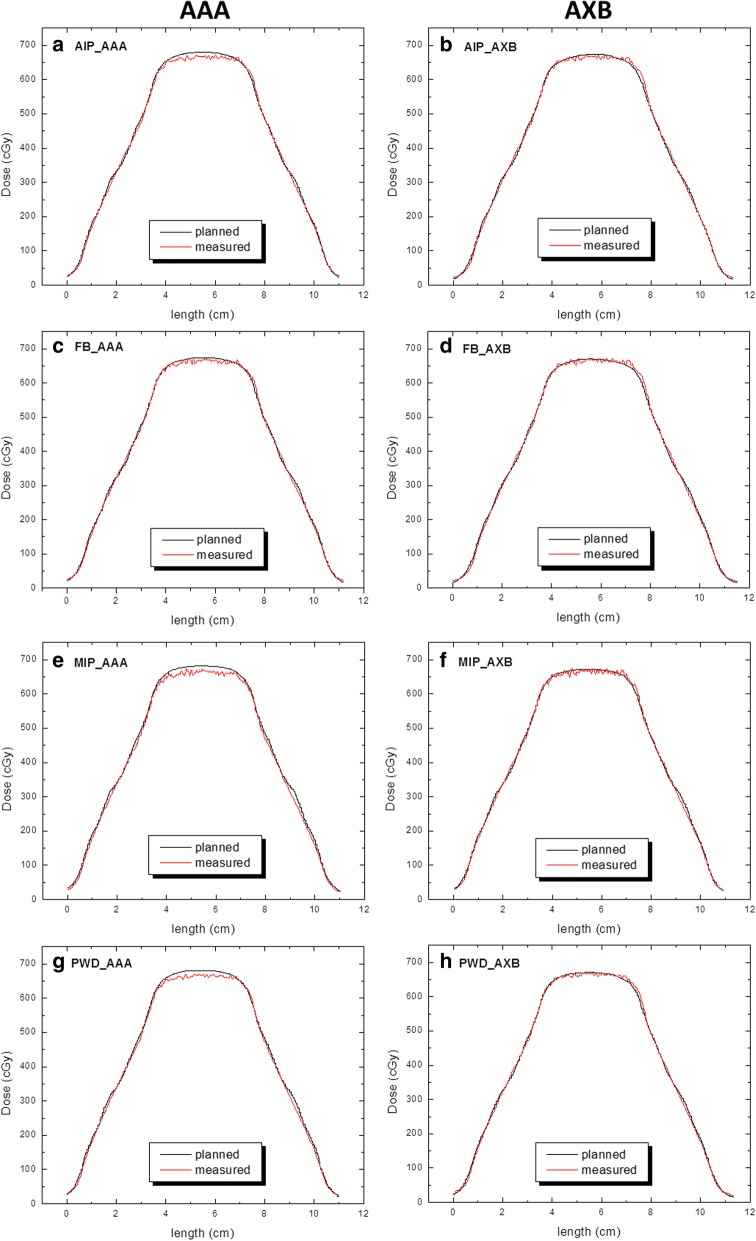
**a**-**h** Dose profiles along the superior/inferior direction of travel as per dataset for AAA and AXB calculations (range of motion = 3 cm). The black curves represent the TPS dose as compared to measured dose indicated by the red curves

A comparison of the dose profiles for 3 cm of motion are shown in Fig. 9a–h. As seen in the left column, the TPS calculated dose for AAA is consistently overestimated with respect to that being measured, and more prominent with respect to MIP and PWD based computations. On the other hand, in the right column the AXB profiles show better conformance with TPS calculated versus measured dose, which give rise to higher pass rates.

## Discussion

Motivation for this research was inspired by a related virtual slab phantom study performed by Aarup. He first reported a large discrepancy between MC and Pencil Beam Convolution (PBC) based dose computations which systematically depended on the density of the surrounding lung tissue [30]. In other previous studies comparing alternative CT data sets, Huang et al. compared the dosimetric accuracy for AIP and MIP projection images for regular and irregular breathing motion [22], Han et al. compared geometric center differences for helical (FB) and AIP image sets [23], and Tian et al. reported on small but significant dosimetric differences between FB, MIP and AIP CT datasets [24]. In many respects, our study is more similar with Oechsner et al. who compared the same plans copied from AIP to FB, MIP and MidV datasets. These datasets were recalculated using the same monitor units to ensure the differences in dose were isolated to the density differences as defined by each dataset [25]. Although each of these previous studies used a single algorithm to compare the differences between datasets to one-another, Oechsner found that the greatest dose differences were between the MIP and FB with D_95%_ ≤ 2.5% when using AAA. Similarly, we observed our greatest dose difference was between MIP and FB datasets with D_98%_ ≤ 2.9% when using AXB. Nonetheless, we are general agreement with findings from another related study by Kroon et al. who compared volumetric modulated (VMAT) plans calculated for AXB and AAA. In Kroon’s study, greater dose differences between algorithms occur with D_98%_ (− 3.2% average), with respect to smaller differences in D_mean_ (− 0.6% average) [31].

In other density related studies, Wiant et al. discovered plans which incorporated a density override region between the ITV and PTV margin [26]. These plans provided more accurate dose modeling and decreased normal lung irradiation for lung SBRT. Interestingly, Liu et al. suggested the tolerance value of the CT number for lung material was ±20 HU in order to keep the associated dose uncertainty at 2–3% [29]. These results are in contrast with our current study where neither AAA nor AXB algorithms predicted a dose error at isocenter to be greater than 2% over a target density variation spanning a delta on the order of 200 HU. More recently, in a 20 patient study conducted by Zvolanek et al. treatment plans based on FB image sets were compared with AIP plans calculated for PBC, AAA, AXB and Voxel Monte Carlo (VMC) algorithms [27]. Even though their computations were done using multiple treatment planning systems, they found dose-differences to be small for Type C computations, so concluded FB and AIP image sets were essentially clinically equivalent. In retrospect, the number of studies directly concerning target density are relatively limited [26, 27, 29, 30]. Furthermore, these studies occasionally involve patient specific datasets where the dosimetric impact is fundamentally tied to the size of the delineation contour as defined by image set.

To the best of our knowledge, ours is the first systematic study to use a combination of 4DCT datasets and algorithms to best represent the temporal density dependence associated with lung tumor movement over an extended range of target motion in a representative heterogeneous phantom. Unlike previous virtual phantom studies, which are usually based on slab geometries to highlight worst case scenarios, this investigation concentrated on using a heterogeneous phantom to better replicate actual density variabilities in order to be more clinically applicable. We also introduced a phase weighted density technique, as an alternative to standard FB and AIP image sets most commonly used for lung dose calculations, and compared these datasets to treatment plans generated using MIP image set. In general, the dose error between AAA and AXB algorithms is small and more prominent when comparing plans generated using higher density MIP image sets. Nonetheless, when subject to stringent SBRT tolerances (gamma 3%, 1 mm), we found marginal discrepancies between datasets representing extreme degrees of tissue heterogeneity for plans calculated with the AXB algorithm. In all cases, however, a reduction in the calculated dose to the lung along with improved delivery accuracy was observed when using AXB over AAA as verified in previous studies [17, 31–33]. Since present organs at risk constraints (e.g. lung, etc.) have been determined using second generation computational algorithms, the “evidence based medicine” nature requires appropriate correction in the lung dose limits to be made before implementing Acuros XB.

Certain limitations of this study include irregular breathing cycles and target motion into the third dimension which would affect target density distributions. In this investigation we minimized the trajectory variables in order to highlight the most clinically relevant scenarios while taking into consideration target densities associated with FB versus MIP datasets. These image sets represent the lowest versus highest density case scenarios, for which the inclusion of 3D motion and/or irregular breathing cycles would only yield a resulting density variation of somewhere in-between. Another limitation is only sparse sampling [10% phase increments] were used in this study. However, as per our experience, this is the normal sampling which is employed by our hospital, as well in a majority of worldwide clinics as it provides a best compromise of dose delivery accuracy along with acceptable work flow efficiency. Moreover, our motion has been very reproducible, and thus we felt 10% phase increments to be sufficient. At our site and at many others sites around the world, inconsistent breathing peaks are minimized by providing patients with audio/video feedback of their respiratory trace which seems to work very well. Excessive care should be taken when dealing with a sudden sneeze or cough which may result in involuntary breathing peaks compromising the quality of the entire 4DCT dataset.

Furthermore, we did not consider direct Monte Carlo dose computation in our comparison. However, in the retroactive study conducted by Zvolanek who evaluated 20 lung cancer patients, they found volumetric Monte Carlo (VMC) computation yielded similar results as AXB and concluded FB and AIP to be clinically equivalent for dose computation in the Monte Carlo era. With the results presented in this study, we have further corroborated the Zvolanek findings since our film analysis suggest the dosimetric discrepancies between FB, AIP, PWD and even MIP dataset indicate minimal favorability over the other when using Type C (MC and AXB) algorithms.

Arguably, although the PWD technique in its current development may be too time consuming to be implemented into clinical practice, it has been demonstrated to be a viable alternative, yielding a delivery accuracy comparable to that generated using the AIP and FB image sets. Additionally, it is noted many clinics have adopted the MIP image set as a way to define the ITV structure and have gone on to calculate dose using AIP of FB datasets. From the results presented in our study, dose computation using Type C algorithms show the dose discrepancies between MIP as compared to FB or AIP may be clinically acceptable. Furthermore, considering the photon attenuation dependence associated with Type C algorithms, an additional reduction of dose could be achieved when using the MIP image set for computation, although further investigation is needed.

## Conclusions

In this heterogeneous phantom study, we evaluated lung target motion ranging up to 3 cm of motion using four distinct CT datasets and two dose algorithms. Despite the substantial density variations between computational datasets over an extensive range of target movement, the dose difference between CT datasets is small and could not be quantified with ion chamber. Radiochromatic film analysis suggests the optimal CT dataset is dependent on the dose algorithm used for evaluation. AIP and FB used with AAA resulted in the best conformance between “measured” verses “calculated” dose for target motion ranging up to 3 cm under both conventional and SBRT tolerance criteria. With AXB, pass rates improved for all datasets with the PWD technique demonstrating slightly better conformity over AIP and FB based computations (gamma 3%, 1 mm). As verified in previous studies, our results confirm a clear advantage in terms delivery accuracy and relative decrease in calculated lung dose when using Acuros XB over AAA. Great care has to be taken when adopting AXB in clinical practice as computed dose differences to various organs need to be correlated with the respective clinical results.

## Abbreviations

4DCT: Four dimensional computed tomography
AAA: Analytical Anisotropic Algorithm
AIP: Average intensity projection
AXB: Acuros External Beam
FB: Free breathing
GTV: Gross tumor volume
ITV: Internal target volume
MC: Monte Carlo
MIP: Maximum intensity projection
PTV: Planned target volume
PWD: Phased weighted density
RTOG: Radiation Therapy Oncology Group
SBRT: Stereotactic body radiation therapy
TPS: Treatment planning system

## References

[1] Wulf J, Haedinger U, Oppitz U, Thiele W, Mueller G, Flentje M (2004). Stereotactic radiotherapy for primary lung cancer and pulmonary metastases: a noninvasive treatment approach in medically inoperable patients. Int J Radiat Oncol Biol Phys.

[2] Benedict SH, Yenice KM, Followill D (2010). Stereotactic body radiation therapy: the report of AAPM task group 101. Med Phys.

[3] Bethesda M (1989). Tissue substitutes in radiation Dosimetry and measurement.

[4] Keall PJ, Mageras GS, Balter JM (2006). The management of respiratory motion in radiation oncology report of AAPM Task Group 76a. Med Phys.

[5] Onishi H, Kuriyama K, Komiyama T (2003). CT evaluation of patient deep inspiration self-breath-holding: how precisely can patients reproduce the tumor position in the absence of respiratory monitoring devices?. Med Phys.

[6] Mampuya WA, Nakamura M, Matsuo Y (2013). Interfraction variation in lung tumor position with abdominal compression during stereotactic body radiotherapy. Med Phys.

[7] Ling CC, Yorke E, Fuks Z (2006). From IMRT to IGRT: frontierland or neverland?. Radiother Oncol.

[8] Gierga DP, Brewer J, Sharp GC (2005). The correlation between internal and external markers for abdominal tumors: implications for respiratory gating. Int J Radiat Oncol Biol Phys.

[9] Radiation Therapy Oncology Group (2009). RTOG 0915: a randomized phase ii study comparing 2 stereotactic body radiation therapy (SBRT) schedules for medically inoperable patients with stage I peripheral non-small cell lung cancer.

[10] Mexner V, Wolthaus JW, van Herk M, Damen EM, Sonke JJ (2009). Effects of respiration-induced density variations on dose distributions in radiotherapy of lung cancer. Int J Radiat Oncol Biol Phys.

[11] Vanderstraeten B, Reynaert N, Paelinck L (2006). Accuracy of patient dose calculation for lung IMRT: a comparison of Monte Carlo, convolution/superposition, and pencil beam computations. Med Phys.

[12] Ding GX, Duggan DM, Lu B (2007). Impact of inhomogeneity corrections on dose coverage in the treatment of lung cancer using stereotactic body radiation therapy. Med Phys.

[13] Miura H, Masai N, Oh RJ, Shiomi H, Yamada K, Sasaki J, Inoue T (2014). Clinical introduction of Monte Carlo treatment planning for lung stereotactic body radiotherapy. J Appl Clin Medical Phys.

[14] Chen WZ, Xiao Y, Li J (2014). Impact of dose calculation algorithm on radiation therapy. World J Radiol.

[15] Ojala JJ, Kapanen MK, Hyödynmaa SJ, Wigren TK, Pitkänen MA (2014). Performance of dose calculation algorithms from three generations in lung SBRT: comparison with full Monte Carlo-based dose distributions. J Appl Clin Med Phys.

[16] Van Esch A, Tillikainen L, Pyykkonen J (2006). Testing of the analytical anisotropic algorithm for photon dose calculation. Med Phys.

[17] Bush K, Gagne IM, Zavgorodni S, Ansbacher W, Beckham W (2011). Dosimetric validation of Acuros® XB with Monte Carlo methods for photon dose calculations. Med Phys.

[18] Sievinen J, Waldemar U, Wolfgang K (2005). AAA photon dose calculation model in Eclipse™.

[19] Failla GA, Wareing T, Archambault Y, Thompson S (2010). Acuros XB advanced dose calculation for the eclipse treatment planning system.

[20] Vedam SS, Kini VR, Keall PJ, Ramakrishnan V, Mostafavi H, Mohan R (2003). Quantifying the predictability of diaphragm motion during respiration with a noninvasive external marker. Med Phys.

[21] Rietzel E, Chen GT, Choi NC, Willet CG (2005). Four-dimensional image-based treatment planning: target volume segmentation and dose calculation in the presence of respiratory motion. Int J Radiat Oncol Biol Phys.

[22] Huang L, Park K, Boike T (2010). A study on the dosimetric accuracy of treatment planning for stereotactic body radiation therapy of lung cancer using average and maximum intensity projection images. Radiother Oncol.

[23] Han K, Basran PS, Cheung P (2010). Comparison of helical and average computed tomography for stereotactic body radiation treatment planning and normal tissue contouring in lung cancer. Clin Oncol.

[24] Tian Y, Wang Z, Ge H, Zhang T, Cai J, Kelsey C, Yoo D, Yin FF (2012). Dosimetric comparison of treatment plans based on free breathing, maximum, and average intensity projection CTs for lung cancer SBRT. Med Phys.

[25] Oechsner M, Odersky L, Berndt J, Combs SE, Wilkens JJ, Duma MN (2015). Dosimetric impact of different CT datasets for stereotactic treatment planning using 3D conformal radiotherapy or volumetric modulated arc therapy. Radiat Oncol.

[26] Wiant D, Vanderstraeten C, Maurer J, Pursley J, Terrell J, Sintay BJ (2014). On the validity of density overrides for VMAT lung SBRT planning. Med Phys.

[27] Zvolanek K, Ma R, Zhou C (2017). Still equivalent for dose calculation in the Monte Carlo era? A comparison of free breathing and average intensity projection CT datasets for lung SBRT using three generations of dose calculation algorithms. Med Phys.

[28] Low DA, Harms WB, Mutic S, Purdy JA (1998). A technique for the quantitative evaluation of dose distributions. Med Phys.

[29] Liu Q, Liang J, Stanhope CW, Yan D (2016). The effect of density variation on photon dose calculation and its impact on intensity modulated radiotherapy and stereotactic body radiotherapy. Med Phys.

[30] Aarup LR, Nahum AE, Zacharatou C, Juhler-Nøttrup T, Knöös T, Nyström H, Specht L, Wieslander E, Korreman SS (2009). The effect of different lung densities on the accuracy of various radiotherapy dose calculation methods: implications for tumour coverage. Radiother Oncol.

[31] Kroon PS, Hol S, Essers M (2013). Dosimetric accuracy and clinical quality of Acuros XB and AAA dose calculation algorithm for stereotactic and conventional lung volumetric modulated arc therapy plans. Radiat Oncol.

[32] Fogliata A, Nicolini G, Clivio A, Vanetti E, Cozzi L (2012). Critical appraisal of Acuros XB and anisotropic analytic algorithm dose calculation in advanced non-small-cell lung cancer treatments. Int J of Radiat Oncol Biol Phys.

[33] Huang B, Wu L, Lin P, Chen C (2015). Dose calculation of Acuros XB and anisotropic analytical algorithm in lung stereotactic body radiotherapy treatment with flattening filter free beams and the potential role of calculation grid size. Radiat Oncol.

